# Analysis of the expression and genetic alteration of CLDN18 in gastric cancer

**DOI:** 10.18632/aging.103457

**Published:** 2020-07-15

**Authors:** Jian Li, Yao Zhang, Dengmin Hu, Tuping Gong, Run Xu, Jun Gao

**Affiliations:** 1 Department of General Surgery, The Third Hospital of Mianyang, Sichuan Mental Health Center, Mianyang 621000, Sichuan, China

**Keywords:** CLDN18, gastric cancer, gene expression, biological network, prognosis

## Abstract

Claudin 18 (CLDN18) is a transmembrane protein that localizes to apical regions to form tight junction complexes. Abnormal expression of *CLDN18* has been reported in gastric cancer (GC). The expression, genetic alterations, and prognostic role of *CLDN18* were analyzed using public data from The Cancer Genome Atlas (TCGA), Gene Expression Omnibus (GEO), and Human Protein Atlas (HPA) databases using multiple online tools. The biological network of *CLDN18* was determined using GeneMANIA. Expression of *CLDN18* was restricted to lung and stomach in normal tissues, was significantly downregulated in GC, but was ectopically overexpressed in some other cancer types. There was no correlation between mRNA expression of *CLDN18* and the clinicopathology of GC, although expression was higher in the Epstein-Barr virus (EBV)-positive subgroup than other subgroups. Genetic alteration of *CLDN18* was not a common event in GC; the main alteration was gene fusion with *ARHGAP26*. *CLDN18* expression did not predict the overall survival (OS) of GC patients. This study summarizes the expression features of *CLDN18* in GC and suggests it may serve as a biomarker and therapy target for GC.

## INTRODUCTION

Gastric cancer is one of the most common malignancies and one of the leading causes of cancer-related mortality worldwide [1]. Gastrectomy with D2 lymphadenectomy is the first treatment choice for advanced disease and improves survival [2]. For patients with locally advanced incurable, recurrent, or metastatic GC, chemotherapy with platinum and fluoropyrimidine derivatives is the standard of care [3], but the five year survival rate is less than 5% [4]. Recently, agents targeting antigens expressed on tumor cells (cetuximab, trastuzumab) or in the tumor microenvironment (nivolumab, pembrolizumab, ramucirumab) have been evaluated in patients with GC, and the objective response rates (ORR) ranged between 3% and 11% [5–7]. Therefore, there is a dire need for the identification and characterization of novel molecules that can be exploited for targeted treatment.

An ideal target for antibody-mediated cancer immunotherapy should meet two criteria: positive expression with epitope accessibility in malignant tissue, and restricted or no expression and epitope inaccessibility in normal tissues. The tight junction protein Claudin 18 splicing isoform A2 (Claudin 18 A2) in the stomach has been identified as a promising target for the treatment of GC [8, 9]. The expression of this tetraspanin membrane protein is strictly confined to differentiated cells in gastric mucosa and is absent from stem cell zone of gastric glands. In addition, the membrane of a considerable number of GC cells express Claudin 18 A2 whose epitopes can be targeted by antibodies [8]. Therefore, a chimeric IgG1 monoclonal antibody zolbetuximab (IMAB362) that specifically binds to Claudin 18 A2 has been developed and is currently being tested in clinical trials with promising preliminary results [10, 11]. Different from other targeted therapies against molecules involving in classic signaling pathways, immune checkpoints or cell cycle, which were used to block or activate the function of targeted molecules, zolbetuximab was demonstrated to mediate cancer cell death through antibody-dependent cellular cytotoxicity (ADCC) and complement-dependent cytotoxicity (CDC). Responders in a Phase II study had 70% Claudin 18 A2-positive tumor cells, suggesting a correlation between Claudin 18 A2 expression and therapeutic benefit [11]. However, previous reports described a wide range of expression of Claudin 18 in patients with GC, but this may be due to the different monoclonal antibodies used for detection and various scoring algorithms used in those immunohistochemistry studies. Therefore, we conducted this bioinformatic analysis to determine the expression pattern of Claudin 18 in GC patients in multiple public databases.

## RESULTS

### Differential mRNA expression of the *CLDN* family in GC

**Figure 1:**
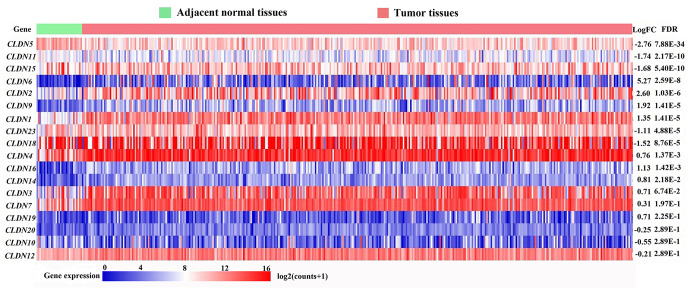
**Heatmap of transcriptional profiles of the *CLDN* family in tumor and adjacent normal tissues from the TCGA-STAD database.** FC, fold change; FDR, false discovery rate.

A total of 407 tissues (375 tumor tissues and 32 adjacent noncancerous tissues) from 381 patients in the Cancer Genome Atlas (TCGA) database based on our search strategies for stomach adenocarcinoma (STAD) (TCGA-STAD cohort) were used to identify the differentially expressed genes (DEGs) of the *CLDN* family. The mRNA expression of 24 *CLDN* family members were determined. The expression of *CLDN8*, *CLDN17*, *CLDN22*, *CLDN24*, *CLDN25*, and *CLDN34* were extremely low and were excluded from analysis. Twelve *CLDN* genes were significantly dysregulated; of these, 10 were considered DEGs based on predefined cutoffs. The expression of *CLDN1*, *CLDN2*, *CLDN6*, *CLDN9*, and *CLDN16* was upregulated, and *CLDN5*, *CLDN11*, *CLDN15*, *CLDN18*, and *CLDN23* downregulated in GC. The log2FC (fold change) of *CLDN18* was -1.52, and the *P* value and false discovery rate (FDR) were 4.38E-05 and 8.76E-05, respectively (Figure 1).

### The mRNA expression of *CLDN18* in various cancers and corresponding normal tissues

**Figure 2:**
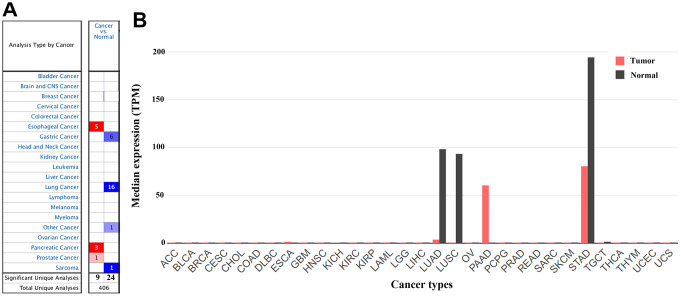
**The gene expression profiles of *CLDN18* across all tumor samples.** (**A**) mRNA expression levels of *CLDN18* in various types of cancer from the Oncomine database. The cell number represents the number of datasets that met the thresholds. The color intensity is proportional to the significance of dysregulation. (**B**) The gene expression profiles of *CLDN18* across all tumor samples and paired normal tissues in the GEPIA2 database. The height of the bar represents the median expression level. TPM, transcripts per kilobase of exon per million mapped reads.

In the Oncomine database, *CLDN18* mRNA expression differences were reported in a total of 406 unique analyses, among which 33 were significant (*P*< 0.05). *CLDN18* expression was downregulated in GC, lung cancer, and sarcoma (gastric stromal tumor) compared to their normal counterparts, whereas upregulation was found in esophageal, pancreatic, and prostate cancer. Six of 20 analyses from six datasets reported downregulated *CLDN18* mRNA expression in GC tissues, while no analysis reported upregulated *CLDN18* mRNA expression (Figure 2A). The results in Gene Expression Profiling Interactive Analysis 2 (GEPIA2) showed that *CLDN18* was strictly expressed in gastric and pulmonary tissues but downregulated in corresponding cancer tissues, although the level was still high in GC compared with other cancers (Figure 2B). In contrast, ectopic overexpression of *CLDN18* was observed in pancreatic cancer.

### Transcription levels of *CLDN18* isoforms

**Figure 3:**
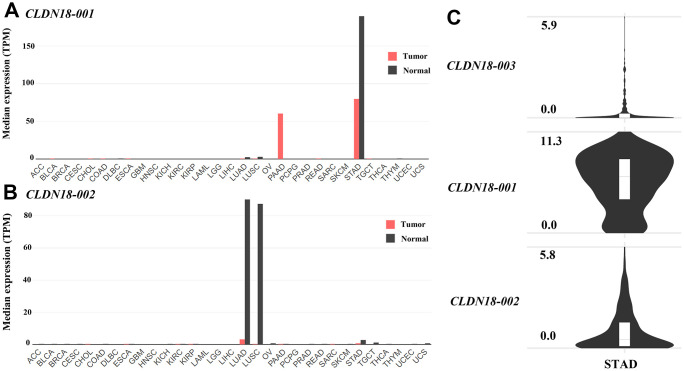
**Transcript levels of isoforms of *CLDN18* in the GEPIA2 database.** (**A**) The transcript levels of *CLDN18-001* across all tumor samples and paired normal tissues. (**B**) The transcript levels of *CLDN18-002* across all tumor samples and paired normal tissues. (**C**) The transcript levels of three isoforms of *CLDN18* in GC tissues. The height of the bars of (**A**, **B**) represents the median expression levels transformed by TPM. The Y axis of (**C**) represents the expression level transformed by log2(counts+1). TPM, transcripts per kilobase of exon per million mapped reads.

Analysis using GEPIA2 showed that *CLDN18-001* (ENST00000343735.8), which encodes isoform 2, also known as isoform A2, was mostly expressed in normal gastric and GC tissues. *CLDN18-001* expression was downregulated in GC compared to normal tissues (Figure 3A). The expression of *CLDN18-002* (ENST00000183605.9), which encodes isoform 1, also known as isoform A1, was restricted to pulmonary normal tissues and was downregulated in lung cancer tissues (Figure 3B). The ectopic expression in pancreatic cancer tissues was mainly *CLDN18-001* (Figure 3A). In GC tissues, the transcript levels of *CLDN18-001* were higher than those of *CLDN18-002* and *CLDN18-003*, the latter being a nonsense mediated decay transcript (Figure 3C).

### The expression changes of *CLDN18* in precancerous tissues of the stomach

**Figure 4:**
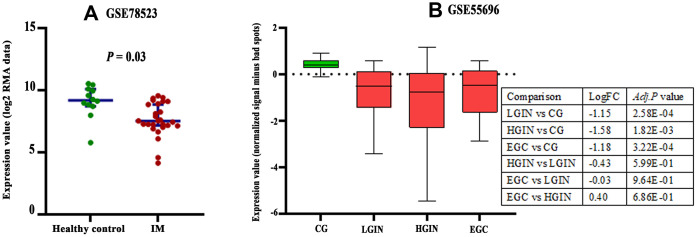
**Expression changes of *CLDN18* in precancerous tissues of the stomach.** (**A**) The difference in *CLDN18* expression between intestinal metaplasia (IM) and healthy controls. (**B**) The difference in *CLDN18* expression among chronic gastritis (CG), low grade intraepithelial neoplasia (LGIN), high grade intraepithelial neoplasia (HGIN), and early gastric cancer (EGC). FC, fold change.

There are three probes in the GSE78523 dataset designed to detect *CLDN18-001* mRNA expression. The median expression was decreased in intestinal metaplasia compared to normal gastric tissues (Figure 4A). In the GSE55696 dataset, *CLDN18* expression was decreased in low grade intraepithelial neoplasia (LGIN), high grade intraepithelial neoplasia (HGIN), and early gastric cancer (EGC) tissues compared to chronic gastritis tissues. No difference was found between LGIN, HGIN, and EGC (Figure 4B).

### Correlation between *CLDN18* expression and clinicopathological characteristics

**Figure 5:**
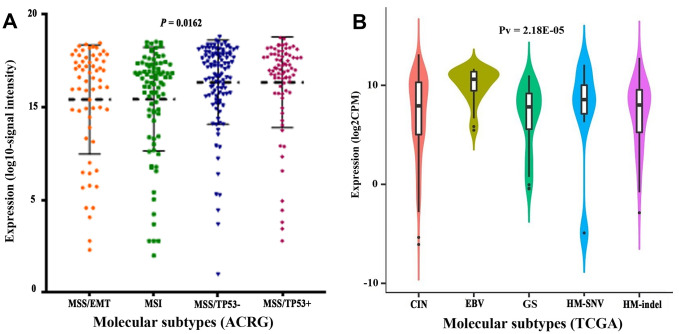
**Transcript levels of *CLDN18* among molecular subtypes.** (**A**) Molecular subtypes of GC according to the Asian Cancer Research Group (ACRG). (**B**) Molecular subtypes of GC according to the Cancer Genome Atlas (TCGA). EMT, epithelial-mesenchymal transition; MSI, microsatellite instability; MSS, microsatellite stable; TP53, tumor protein p53; CIN, chromosomal instability; EBV, Epstein-Barr virus; GS, genomically stable; HM, high mutation; SNV, single nucleotide variants.

We compared clinicopathological characteristics between *CLDN18*-high and *CLDN18*-low groups, with the median expression as cutoff, in the TCGA-STAD cohort. There was no relationship between *CLDN18* expression and age, sex, race, ethnicity, T stage, node metastasis, TNM stage, histological type, or tumor location (Table 1). *CLDN18* expression differences were significant in certain molecular classifications. *CLDN18* expression was higher in the microsatellite stable/p53 positive (MSS/TP53+) and negative (MSS/TP53-) subgroups versus others using the Asian Cancer Research Group (ACRG) classifications (Figure 5A) and in the EBV-positive subgroup versus others using the TCGA classifications (Figure 5B).

**Table 1:** Characteristics of GC patients of *CLDN18*-low and *CLDN18*-high groups in TCGA-STAD.

**Clinicopathological characteristics**	***CLDN18*-low (n=187)**	***CLDN18*-high (n=188)**	**χ^2^**	***P* value**
Age (years)			0.497	0.481
<60	61	55		
≥60	126	133		
Sex			3.107	0.078
Male	112	129		
Female	75	59		
Race			0.895	0.639
Asian	41	33		
Black	5	6		
White	118	120		
T stage			0.148	0.701
T1+ T2	48	51		
T3+ T4	136	132		
Node metastasis			1.290	0.256
Negative	60	51		
Positive	117	129		
TNM stage			0.010	0.921
I+II	82	82		
III+IV	95	93		
Histological type			5.329	0.502
Adenocarcinoma, intestinal type	30	44		
Carcinoma, diffuse type	31	30		
Adenocarcinoma, NOS	69	68		
Mucinous adenocarcinoma	11	8		
Papillary adenocarcinoma, NOS	4	1		
Signet ring cell carcinoma	7	5		
Tubular adenocarcinoma	34	32		
Tumor location			1.672	0.433
Cardia and fundus	62	71		
Body	43	47		
Antrum and pylorus	73	62		

NOS: not otherwise specified.

### *CLDN18* protein expression by immunohistochemistry

**Figure 6:**
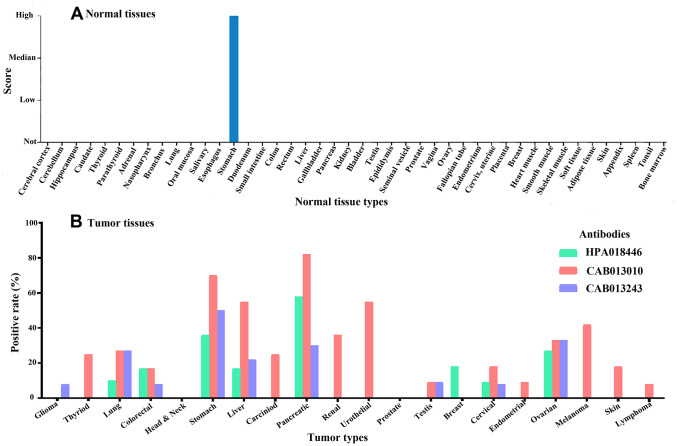
The expression of Claudin 18 in normal (**A**) and tumor (**B**) tissues.

Three antibodies were used to detect Claudin 18. Antibody HPA018446 detects isoforms A2 and A1. The isoforms detected by antibodies CAB13010 and CAB013243 are not known. Expression was only detected in gastric glandular cells in normal tissues (Figure 6A) but was detected in many cancer types, with high expression in gastric, pancreatic, lung, and ovarian cancer tissues (Figure 6B). In GC tissues, Claudin 18 was detected in the cytoplasm and on the membrane. Rates of Claudin 18 expression in published studies are summarized in Table 2.

**Table 2:** Summaries of the positive rates of Claudin 18 in gastric cancer.

**Author or dataset**	**Antibody**	**Samples**	**Positive rate (%)**
HPA [12]	HPA018446	11	18.2
HPA [12]	CAB013010	10	30.0
HPA [12]	CAB013243	12	33.3
MONO study [11]	Polyclonal	268	31.0
Coati I [13]	clone 34H14L15	523	68.8
Rohde C [14]	clone 43-14A	262	52.0
Dottermusch M [15]	clone EPR19202	474	42.2

### Analysis of *CLDN18* genetic alterations

**Figure 7:**
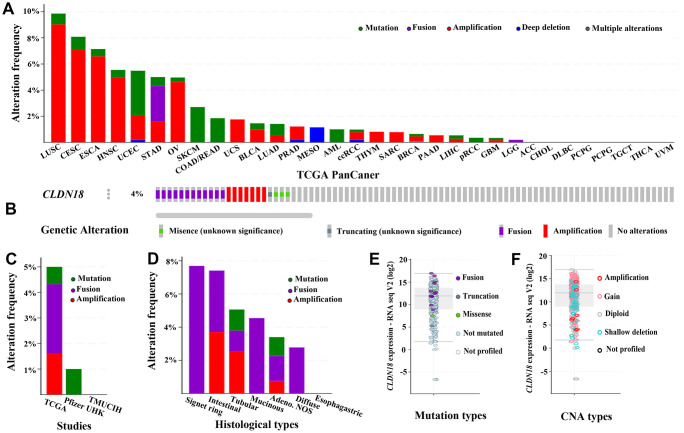
**The genetic alterations of *CLDN18* in cancers.** (**A**) Frequency of genetic alterations in various types of cancer derived from TCGA PanCan datasets. (**B**) OncoPrint visual summary of variations on a query of *CLDN18*. (**C**) Analyses of genetic variations of *CLDN18* reported in different studies. (**D**) Analyses of genetic variations of *CLDN18* reported in different histological types. (**E**) The mRNA expression of mutated *CLDN18*. (**F**) The mRNA expression of *CLDN18* with copy number alterations (CNA).

Genetic alterations of *CLDN18* in different cancers were examined using the TCGA PanCan Atlas studies. Gene amplification mainly occurred in lung squamous cell, cervical, esophageal, head & neck, and ovarian cancer. Mutation predominated in uterine cancer, and gene fusion in stomach cancer (Figure 7A). cBioPortal has seven archived datasets of genetic alterations in human GC; four datasets were excluded due to overlapping original samples. A total of 618 cases of GC from three datasets were included for analysis. Four percent (23/618) of patients were found to have gene alterations: 12 fusions, seven amplifications, one truncating mutation, and three missense mutations (Figure 7B–7D). The main genetic alteration was *CLDN18-ARHGAP26* fusion (9/23). *CLDN18* alterations in GC patients from the TCGA PanCan Atlas data is shown in Figure 7E, 7F.

### Biological interaction network of *CLDN18*

**Figure 8:**
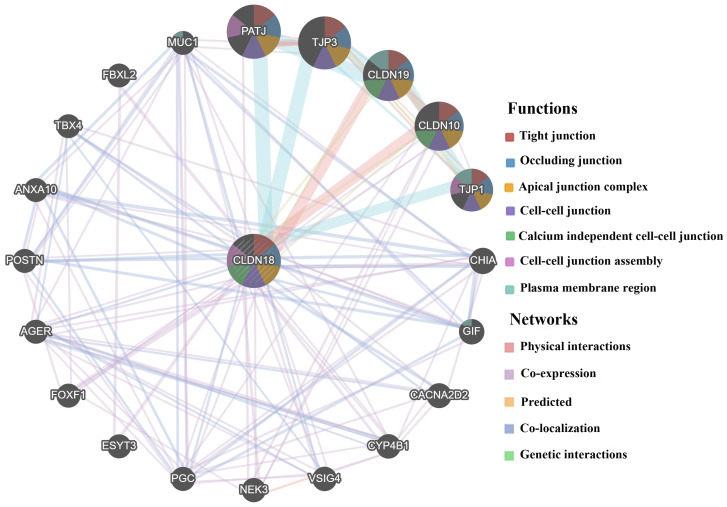
**Biological interaction network of *CLDN18* analyzed using GeneMANIA.**

The results of GeneMANIA showed that *CLDN18* could share protein domains, physically interact with *CLDN* family members *CLDN10* and *CLDN19*, colocalize with 11 proteins, and coexpress with 19 proteins (Figure 8). The top five genes displaying the greatest correlations with *CLDN18* included *CLDN10*, *CLDN19*, *PATJ* (crumbs cell polarity complex component), *TJP1* (tight junction protein 1), and *TJP3* (tight junction protein 3). Further functional analysis revealed that these genes are mainly involved in “cell-cell junction assembly”, especially “tight junction” (FDR: 2.95E-7).

### The prognostic value of *CLDN18* expression

**Figure 9:**
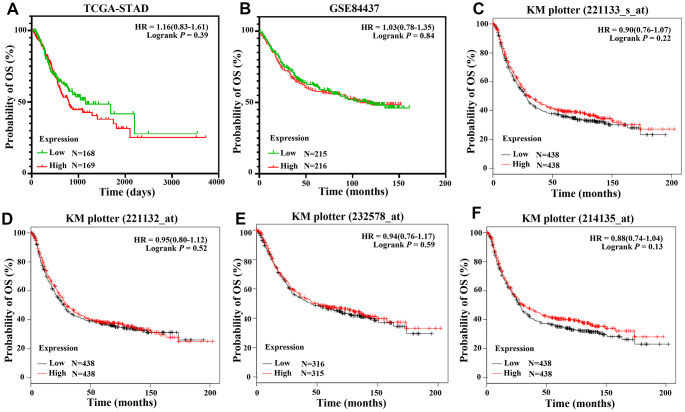
**Overall survival of GC patients grouped by *CLDN18* median cutoff into high and low groups.** (**A**) TCGA-STAD; (**B**) GSE84437; (**C**–**F**) KM-plotter, data from six datasets: GSE14210, GSE15459, GSE22377, GSE29272, GSE52205, GSE62254.

There were 337 and 431 patients with eligible survival data in the TCGA-STAD and GSE84437 datasets, respectively. mRNA values in GSE84437 were detected for *CLDN18-001*. Six datasets with a total of 1051 patients (GSE14210: N=146; GSE15459: N=200; GSE22377: N=43; GSE29272: N=268; GSE52205: N=94; GSE62254: N=300) were used for Kaplan-Meier survival curves. Four probes were used to test the mRNA expression in the six datasets; two were specific for *CLDN18-002* (221132_at, 221133_s_at), while the other two probes (232578_at, 214135_at) were not isoform-specific. All analyses showed that the expression of *CLDN18* is not related to the overall survival of GC patients (Figure 9).

## DISCUSSION

Claudin 18 is a member of a family of at least 27 transmembrane proteins. These proteins are mainly in apical regions forming tight-junction complexes, playing a critical role in cell-cell adhesion, maintenance of cell polarity, and selective paracellular permeability [16–18]. It has two isoforms, which are specific tight junction components of pulmonary and gastric tissues. This was confirmed by GEPIA2 and HPA analysis.

In mouse models, Claudin 18 loss increased H^+^ leakage, inflammatory cell infiltration, and gastric metaplasia [19], resulting in intraepithelial neoplasia and invasive tumors [20]. In human studies, *CLDN18* is downregulated in a subset of GCs [21–23]. These findings suggest that Claudin 18 loss induces gastritis and creates an inflammatory setting for dysplasia and/or cancer. Additionally, loss of Claudin 18 can lead to activation or translocation of some kinases in several pro-oncogenic pathways [24]. These findings lead to important questions regarding the role of Claudin 18 in GC.

Although Claudin 18 loss may be involved in the carcinogenesis of GC, it was retained in some cancer tissues, but with a range of expression across studies (Table 2). Those studies were performed with different antibodies with different sensitivities/specificities, and the results assessed by different scoring systems. This supports the need for testing and scoring standardization. No relationship between *CLDN18* expression and clinicopathological characteristics in the TCGA-STAD cohort was found. This was consistent with Dottermusch et al. [15] but somewhat different from other studies. Coati I et al. found that tumors localized in the gastric corpus and tumors of the diffuse type showed a higher positive rate of Claudin 18 [13]. Claudin 18 A2 expression was also found to be significantly higher in GCs of the diffuse subtype and high grade (G3) in Japanese patients [14]. This may be due to GC heterogeneity, patient ethnicity, and detection methods between the studies.

A relationship between *CLDN18* expression and molecular classification was found in this study. *CLDN18* expression was higher in the EBV-positive subgroup by TCGA classification and in the MSS subgroups by ACRG classification. Because infection with EBV was more frequent in the MSS/TP53+ group, this suggests EBV infection increases *CLDN18* expression. This increase is consistent with three immunohistochemistry studies [13, 15, 25]. EBV-associated GC is a unique etiological entity. Increased Claudin 18 A2 may be a key features of EBV-mediated carcinogenesis. EBV infection of epithelial cells is mediated by cell-to-cell contact, and extensive cell junctions may restrict antibody accessibility to the virus [26, 27]. This suggests a role of Claudin 18 in ensuring EBV maintenance in tumor cells.

Although genetic alteration of *CLDN18* was infrequent in GC, interchromosomal translocation between *CLDN18* and *ARHGAP26* was found in genomically stable tumors by TCGA classification category. Fusion events were enriched in signet-ring cells, mucinous cells, and diffuse-type GC. ARHGAP26 is a GTPase-activating protein (GAP) that induces cellular motility [28]. The fusion conserves the RHO GTPase activating domain of ARHGAP26 but deletes the C-terminal PDZ-binding motif of Claudin 18 which allows Claudin 18 to bind the actin cytoskeleton. Consistent with the fusion protein overexpression [29], mRNA expression of the fusion gene was higher than the median expression of *CLDN18* in patients of the TCGA PanCan cohort. The fusion-positive cancer cells stained diffusely positive for Claudin 18 in addition to membrane staining, suggesting that localization was altered [24]. The contribution of these changes to carcinogenesis remains to be determined.

Matsuda Y et al. reported worse malignancy grades and survival outcomes in GC patients with no expression of Claudin 18 [30]. Two studies with small samples sizes also suggested that reduced Claudin 18 A2 expression correlated with poor prognosis [22, 31]. In contrast, we found no correlation between *CLDN18* expression and survival. This was consistent with the results of a large Caucasian cohort study [15].

## CONCLUSIONS

In normal tissues, *CLDN18* mRNA expression was restricted to the lung and stomach. Although expression was downregulated, it was retained in some GC tissues. Aberrant activation was found in esophageal, pancreatic, ovarian, biliary, and lung adenocarcinomas. Therefore, Claudin 18 may be a candidate biomarker and therapeutic target for these tumors. Divergence in *CLDN18* expression rates across studies may be related to ethnic characteristics or linked to intratumoral GC heterogeneity, which poses a challenge for diagnostic evaluations and targeted therapy. In-depth experiments and well-defined detection approaches are needed to investigate the molecular mechanism, to develop targeted agents, and to screen for patients suited for treatment.

## MATERIALS AND METHODS

### Gene expression data from TCGA and differential expression analysis

The gene expression levels of the *CLDN* family were obtained from the TCGA data portal (https://portal.gdc.cancer.gov/; accessed January 05, 2020) [28]. Relevant search parameters were used as follows: data category: transcriptome profiling; data type: gene expression quantification; experimental strategy: RNA-Seq; workflow type: HTSeq-counts; and project: TCGA-STAD. Differential expression analysis was conducted between tumor and adjacent normal tissues using the R language package EdgeR [32]. To ensure that the expression distributions of each sample were similar across the entire matrix, gene expression levels were normalized by the calcNormFactors function [33]. Log2FC, associated adj. *P* values, and FDRs were calculated. DEGs of the *CLDN* family were identified by using the settings |log2FC| > 1 and adj. *P*<0.05 was used as the cutoffs. We used the log2(counts+1) transformation to convert the expression levels of *CLDNs* for further analysis.

### Analysis of *CLDN18* mRNA expression in different cancers and normal tissues

Analysis of *CLDN18* mRNA expression in different cancers and normal tissues was carried out using Oncomine (https://www.oncomine.org/resource/main.html) [34] and GEPIA2 (http://gepia2.cancer-pku.cn/index.html) [35]. The thresholds for analysis in the Oncomine database were as follows: *P* value: 0.05; FC: 2; gene ranking: 10%; analysis type: cancer vs. normal; and data type: mRNA. The online database GEPIA2 is an interactive web-based tool that includes normal and tumor samples from the Genotype-Tissue Expression (GTEx) projects and TCGA for analyzing RNA sequencing expression data. The database was used to confirm the expression of *CLDN18* in different cancer and normal tissues. The expression of isoforms of *CLDN18* was also determined using GEPIA2. Bar plots were generated to visualize the relationship.

### Changes in the expression of *CLDN18* in precancerous stomach tissues

*Gene Expression Omnibus* (GEO) (https://www.ncbi.nlm.nih.gov/geo/) is a worldwide data repository that distributes gene expression data, including microarray, next-generation sequencing, and other forms of high-throughput functional genomics data. The terms “gastric or stomach” and “cancer” or “tumor” or “carcinoma” or “neoplasm” were used as the search parameters in the GEO database. “*Homo sapiens*” was used to limit the search range. The processed expression data of *CLDN18* were obtained from GSE78523, which includes 30 intestinal metaplasia and 15 adjacent normal gastric tissues [36], and GSE55696, which includes 19 LGIN, 20 HGIN, 19 EGC, and 19 chronic gastritis tissue samples [37]. The differential expression of *CLDN18* was analyzed with GEO2R. When more than one probe was available, the median value was used.

### Correlation between *CLDN18* expression and clinicopathological characteristics

The currently available clinical data of the patients included in this study were downloaded from TCGA, and the correlation between *CLDN18* expression and clinicopathological characteristics was analyzed. The expression difference among molecular subtypes based on ACRG was analyzed using data downloaded from GENT2 (http://gent2.appex.kr/gent2/) [38]. The expression difference among molecular subtypes based on TCGA was analyzed by TISIDB (http://cis.hku.hk/TISIDB) [39].

### Protein expression of *CLDN18* by immunohistochemistry

The Human Protein Atlas (HPA) (https://www.proteinatlas.org) website containing immunohistochemistry data from tissue microarray-based analyses of major cancer tissue types and normal tissues was used [12]. Moderate and strong intensity of staining was considered positive. The positive rates of Claudin 18 in GC from the HPA were summarized together with data from articles available on PubMed [11, 13–15].

### Identification of genetic alterations of *CLDN18* in GC

The cBioPortal (https://www.cbioportal.org/) website of cancer genomics datasets was used [40]. Mutations and copy number alterations (CNAs) of *CLDN18* in GC were analyzed using the cBioPortal tool. The OncoPrint sub-tool was utilized to display an overview of the integrated status of genetic alterations for *CLDN18*. The Cancer Types Summary sub-tool showed the details of genetic alterations in different datasets and in different histological types.

### Identifying the *CLDN18* biological network

GeneMANIA (http://genemania.org/), a web interface, was used to construct a biological network for *CLDN18* in terms of physical interaction, coexpression, colocalization, prediction, and shared protein domains, and evaluate the functions of network components [41]. A figure where nodes symbolize genes and links represent networks was used to display interactions.

### The prognostic value of *CLDN18*

The prognostic value of *CLDN18* expression was determined in the TCGA-STAD cohort and the GSE84437 dataset, which has the largest number of GC patients in GEO. Patients with a follow-up or survival time of less than 1 month were excluded. The Kaplan-Meier plotter online database (http://kmplot.com) was used to validate the relationship between *CLDN18* expression and overall survival (OS) in GC patients [42]. The median value of mRNA expression was used as the cutoff to divide patients into high and low expression groups. Hazard ratios (HRs) with 95% confidence intervals (CIs) and log-rank *P* values were calculated. A *P value* <0.05 was considered statistically significant.

### Statistical analysis

Online analyses were conducted following the statistical methods used by individual bioinformatic websites, and the corresponding parameters described above. Categorical variable numbers (n) are presented. Significant differences among groups were determined using the Pearson χ^2^ test and Mann-Whitney’s U test. The DEG analysis was conducted by R software 3.6.2 (https://www.r-project.org/). Other statistical analyses were performed using IBM SPSS Statistics version 22.0 (IBM Co., Armonk, NY, USA). The graphs, survival curves, and log-rank tests were completed in GraphPad Prism 8.0 (GraphPad Software, San Diego, CA, USA). For all analyses, a two-sided significance level of 0.05 was assumed.

### Ethical statement

This study was approved by the Academic Committee of the Third Hospital of Mianyang and conducted according to the principles expressed in the Declaration of Helsinki. All information in this study was retrieved from public datasets; therefore, written informed consent was not necessary. This study meets the publication guidelines provided by the individual public datasets.
